# The blunted effect of glucose-dependent insulinotropic polypeptide in subcutaneous abdominal adipose tissue in obese subjects is partly reversed by weight loss

**DOI:** 10.1038/nutd.2016.15

**Published:** 2016-05-02

**Authors:** M Asmar, N Arngrim, L Simonsen, A Asmar, P Nordby, J J Holst, J Bülow

**Affiliations:** 1 Department of Clinical Physiology/Nuclear Medicine, Bispebjerg University Hospital, Copenhagen, Denmark; 2 NNF Center for Basic Metabolic Research, University of Copenhagen, Copenhagen, Denmark; 3 Department of Biomedical Sciences, University of Copenhagen, Copenhagen, Denmark

## Abstract

**Background:**

Glucose-dependent insulinotropic polypeptide (GIP) appears to have impaired effect on subcutaneous abdominal adipose tissue metabolism in obese subjects. The aim of the present study was to examine whether weight loss may reverse the impaired effect of GIP on subcutaneous abdominal adipose tissue in obese subjects.

**Methods:**

Five obese males participated in a 12-week weight loss program, which consisted of caloric restriction (800 Cal day^−^^1^) followed by 4 weeks of weight-maintenance diet. Before and after weight loss, subcutaneous adipose tissue lipid metabolism was studied by conducting regional measurements of arterio-venous plasma concentrations of metabolites and blood flow (adipose tissue blood flow, ATBF) across a segment of the abdominal adipose tissue in the fasting state and during GIP infusion (1.5 pmol kg^−^^1 ^min^−^^1^) in combination with a hyperinsulinemic–hyperglycemic clamp.

**Results:**

After weight loss (7.5±0.8 kg), glucose tolerance and insulin sensitivity increased significantly as expected. No significant differences were seen in basal ATBF before (1.3±0.4 ml min^−1^ 100 g tissue^−1^) and after weight loss (2.1±0.4 ml min^−1^ 100 g tissue)^−1^; however, a tendency to increase was seen. After weight loss, GIP infusion increased ATBF significantly (3.2±0.1 ml min^−1^ 100 g tissue^−1^) whereas there was no increase before weight loss. Triacylglycerol (TAG) uptake did not change after weight loss. Baseline free fatty acid (FFA) and glycerol output increased significantly after weight loss, *P<*0.001. During the clamp period, FFA and glycerol output declined significantly, *P<*0.05, with no differences before and after weight loss. Weight loss increased glucose uptake and decreased FFA/glycerol ratio during the clamp period, *P<*0.05.

**Conclusions:**

In obese subjects, weight loss, induced by calorie restriction, improves the blunted effect of GIP on subcutaneous abdominal adipose tissue metabolism.

## Introduction

In healthy normal-weight subjects, subcutaneous abdominal adipose tissue blood flow (ATBF) increases from two- to three-fold in response to nutritional stimuli,^1,2^ suggesting that a link may exist between the gastrointestinal tract and ATBF. In a recent study, we showed that the incretin hormone glucose-dependent insulinotropic polypeptide (GIP) may regulate ATBF.^3^ GIP in combination with a hyperinsulinemic–hyperglycemic clamp — with plasma glucose and insulin concentrations similar to those found after ingestion of a carbohydrate-rich meal — increased ATBF, resulting in an increase in triglyceride (TAG) deposition in the subcutaneous abdominal adipose tissue.^3^ These effects seem to be beneficial, as efficient fat deposition in the subcutaneous adipose depots protects other tissues from potentially hazardous, prolonged exposure to high levels of lipids postprandially.

Both fasting ATBF and its responsiveness to nutrients are reduced in obese individuals.^4^ It has been shown that ATBF is suppressed after meal ingestion at all stages of glucose intolerance, including T2DM.^5^ Obese insulin-resistant subjects display lower insulin-stimulated glucose uptake and lower blood flow responses in both visceral and subcutaneous abdominal fat compared with lean subjects.^6^ Recently, we investigated the effects of GIP in combination with hyperinsulinemia and hyperglycemia on ATBF and subcutaneous abdominal adipose tissue metabolism in obese subjects with either normal glucose tolerance (NGT) or impaired glucose tolerance (IGT).^7^ A small but significant vasodilatation took place in adipose tissue in the obese subjects with NGT, whereas it was nearly absent in the subjects with IGT. The vasodilatation was only transient compared with the prolonged and sustained vasodilatation, we found in healthy lean subjects during GIP and insulin infusion.^3^ Furthermore, the transient increase in ATBF in the obese subjects with NGT did not affect the adipose tissue metabolism in contrast to the effect in healthy lean subjects, suggesting that the blunted ATBF response in obese subjects is related to insulin/GIP resistance. Weight loss through caloric restriction is known to improve and even restore insulin sensitivity.^8,9^ To our knowledge, only a few studies have reported the effects of weight loss on ATBF and with conflicting results. Weight loss in response to a hypocaloric and to a very-low-calorie diet enhanced ATBF in the short term.^10^ In contrast, it was shown recently that rapid weight loss in obese subjects on very-low-calorie diet induced no modification of ATBF when expressed per gram of adipose tissue mass.^11^ The latter finding is in agreement with a recent publication from our laboratory.^12^

In the present study, we hypothesize that weight loss through caloric restriction reverses GIP resistance and restores the ATBF response in the subcutaneous abdominal adipose tissue. Therefore, the aim of the present study was to study the effect of GIP with hyperinsulinemia and hyperglycemia on subcutaneous abdominal adipose tissue metabolism in obese subjects with IGT before and after weight loss through caloric restriction.

## Subjects and methods

### Subjects

Sample size was determined before the study using power analysis (*P*=0.8, *α*=0.05) with regard to ATBF response based on our two previous publications.^3,7^ Assuming an ATBF response as found in normal-weight, healthy subjects, less than 6 were needed in the present study. Assuming an ATBF response as found in overweight subjects with NGT, 7 were needed in the present study. Therefore, we initially recruited eight obese (body mass index (BMI) >30 kg m^−2^) male subjects with IGT (fasting glucose 5.6–7 mm and 2-h glucose between 7.8 and 11.1 mm) from our previous study.^7^ However, two of the subjects dropped out during the run-in period and one during the weight loss program due to the complexity of the experimental setup and the duration of the weight loss program.

During the run-in period, the subjects were informed not to change their diet and exercise behaviors for 4 weeks before the intervention. They were also asked to refrain from taking weight loss medication and not to change their exercise behaviors during the intervention. Consent to participate was obtained after the subjects had read a description of the experimental protocol, which was approved by the Scientific Ethics Committee of the capital region of Copenhagen.

### Study design and intervention

#### Weight loss study

The subjects participated in a structured, outpatient weight-loss program. To calculate energy intake and macronutrient breakdown, each participant's self-recorded dietary intake was evaluated before the intervention. Recording of food intake was conducted for a 4-day period including 3 weekdays and either Saturday or Sunday. Energy, and macro- and micronutrients content of the diets were determined using http://www.madlog.dk. The subjects were individually counseled by the study dietitian/coordinator before the intervention to consume an 800-kcal deficit diet designed to induce 0.5–1 kg weight loss per week (5–10% total weight loss over 12 weeks). The sessions included adjustments of caloric intake and behavioral therapy. Food diaries were reviewed every third week, and the subjects were given instructions on food intake based on the prescribed energy intake. The subjects were instructed to measure their weight daily. At the end of the weight loss phase, subjects underwent weight-maintenance period of 4 weeks.

The subjects were instructed not to participate in strenuous exercise sessions and not to change their exercise and other physical activities during the period of the study.

#### Anthropometry

Subject's height and weight were measured at baseline and after the 12-week intervention, Table 1. Body composition was determined at baseline and at the end of the 12 weeks by total-body dual-energy X-ray scanning (Lunar DPX-IQ), Table 1. The subcutaneous adipose tissue thickness was measured on the total-body dual-energy X-ray scan in order to estimate whether the dietary treatment resulted in significant changes in the subcutaneous abdominal adipose tissue mass. This was done manually at the horizontal level with the largest subcutaneous thickness between the upper iliac crest and the umbilicus. This position (based on bone markers) was also employed for the post weight loss scans. The delineation of the subcutaneous tissue and the underlying abdominal muscle was defined as a clear shift in the gray scale on the scan.

#### Experimental day

The subjects were instructed to abstain from drinking alcohol during 48 h before the study. On the day of the experiment, subjects arrived at the department at 0900 h, having used non-strenuous transportation and after having fasted for at least 12 h. The investigations were performed with the subjects in supine position in a room kept at 24 °C. After catheterizations as described below, baseline measurements (time=−30, −15 and 0 min) were commenced. Thereafter, a continuous infusion of GIP (1.5 pmol kg^−1^ min^−1^) in combination with a hyperinsulinemic (7mU m^−2^ min^−1^) and a hyperglycemic (7 mm) clamp was initiated at time=0 and continued for 180 min.

#### Catheterization

One catheter (BD Venflon, PRO, Becton Dickinson, Singapore) was inserted into an antecubital vein for the infusion of GIP, glucose and insulin. The subjects were then catheterized in a subcutaneous vein on the anterior abdominal wall and in a radial artery.

A vein draining the subcutaneous abdominal adipose tissue on the anterior abdominal wall was catheterized antegradely as previously described^13^ during ultrasound/color-Doppler imaging of the vein. A 22-g 10-cm polyurethane catheter (Arrow International, Reading, PA, USA) was inserted using the Seldinger technique. The tip of the catheter was positioned above the inguinal ligament in order to minimize the risk of withdrawing blood from the femoral vein. After insertion, the catheter was kept patent throughout the experiment by continuous infusion of saline at a rate of 40 ml h^−1^. Another catheter was inserted percutaneously into the radial artery of the non-dominant arm under local analgesia (1 ml 1% lidocaine) with an Artflon (Becton Dickinson, Erembodegem, Belgium). The catheter was kept patent by regular flushing with saline.

#### Measurements

ATBF measurements were performed continuously for the duration of the experiment by recording washout of ^133^Xenon, which was injected in gaseous form in the adipose tissue. This technique has previously been validated in our laboratory.^13^ About 1 MBq gaseous ^133^Xenon (The Hevesy Laboratory, Risoe National Laboratory, Roskilde, Denmark) mixed in about 0.1 ml of atmospheric air was injected into the para-umbilical area of the subcutaneous adipose tissue. The washout rate of ^133^Xenon was measured by a scintillation-counting device (Mediscint, Oakfield Instruments, Oxford, UK).

#### Blood samples and analysis

Blood samples were drawn at time −30, −15, 0, and hereafter every 30 min until discontinuation of the infusion. Plasma glucose concentrations were measured every 5–10 min for clamp adjustments.

Blood samples were drawn simultaneously from the two catheters for measurements of TAG, glycerol, free fatty acid (FFA) and glucose. In addition, blood was collected from the artery for measurements of GIP, insulin and C-peptide.

Blood samples were collected into ice-chilled tubes (Vacuetta; Greiner Labortechnic, Kremsmünster, Austria). Tubes for intact GIP contained EDTA and a specific DPP-4 inhibitor (valine-pyrrolidide; 0.1 μmol l^−^^1^, Novo Nordisk A/S, Bagsværd, Denmark), and tubes for insulin and C-peptide contained heparin. Tubes for glucose, TAG, FFA and glycerol contained EDTA. All samples were centrifuged within 15 min for 15 min at 5000 *g* at 4 °C and stored at −80 °C until analysis.

Intact GIP was measured as described in Deacon *et al.*^14^ The assay is specific for the intact N-terminus of GIP, the presence of which is required for biological activity of the peptide.

Plasma glucose (Glucose/HK, Roche Diagnostics, Mannheim, Germany), TAG (Triglyceride GPO-PAP, Roche Diagnostics), FFA (NEFA C kit, Wako Chemicals, Neuss, Germany) and glycerol (Boehringer Mannheim, Mannheim, Germany) were measured by enzymatic methods modified to run on a Hitachi 912 automatic analyzer (Boehringer Mannheim).

### Calculations and statistical analysis

The ATBF was calculated from the mean washout rate constant determined in 30-min periods corresponding to the time points when blood samples were drawn. ATBF was then calculated according to the equation ATBF=−*k* × *λ* × 100. A tissue/blood partition coefficient (*λ*) for Xenon of 10  ml g^−1^ was used.^15^

Subcutaneous abdominal adipose tissue metabolic net fluxes were calculated by multiplication of the arterial–venous or venous–arterial concentration differences of the metabolites and the appropriate flow value (whole blood for calculation of glycerol and glucose fluxes^16^ and plasma flow for calculation of fatty acid and TAG fluxes).

All results are presented as mean±s.e.m. Area under the curve was calculated using the trapezoidal rule and are presented as the incremental values (baseline levels subtracted). The significance of changes in ATBF, arterial hormone and metabolite concentrations with time before and after the weight loss was tested using two-way analysis of variance with repeated measures. Significant differences in ATBF area under the curve and metabolite fluxes before and after weight loss were evaluated by paired *t*-test. *P-*values of <0.05 were considered as statistically significant.

## Results

### Changes in body weight and body composition

**Figure 1:**
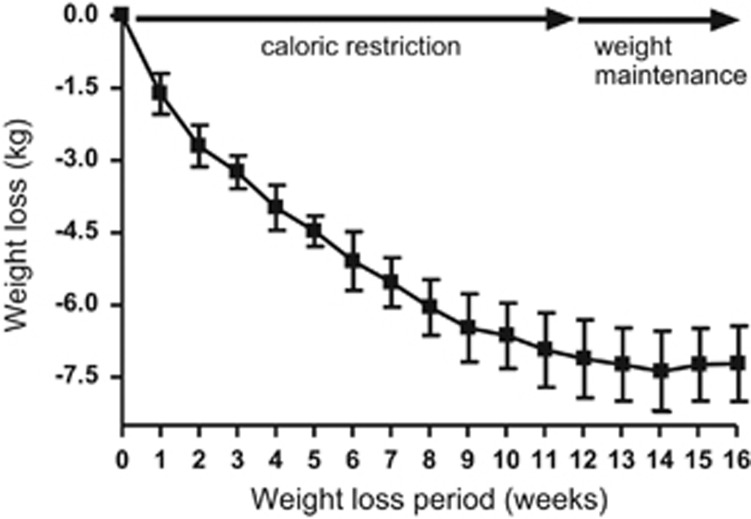
Progression of weight loss during 12 weeks of caloric restriction followed by 4 weeks of weight stabilization.

Mean weight loss was 7.5±0.8 kg, representing about 7.5% decrease in body weight. As shown in Figure 1, weight loss occurred rapidly at the start of the weight loss program and was progressive during the initial 12 weeks and then, as was intended by the study design, weight was maintained at stable levels for 4 weeks before the follow-up metabolic studies. BMI, % body fat and fat mass also declined significantly, Table 1. The mean absolute calorie intakes before and after weight-maintenance period were 2252±294 and 1512±96, respectively.

### Changes in arterial hormone and metabolite concentrations

**Figure 2:**
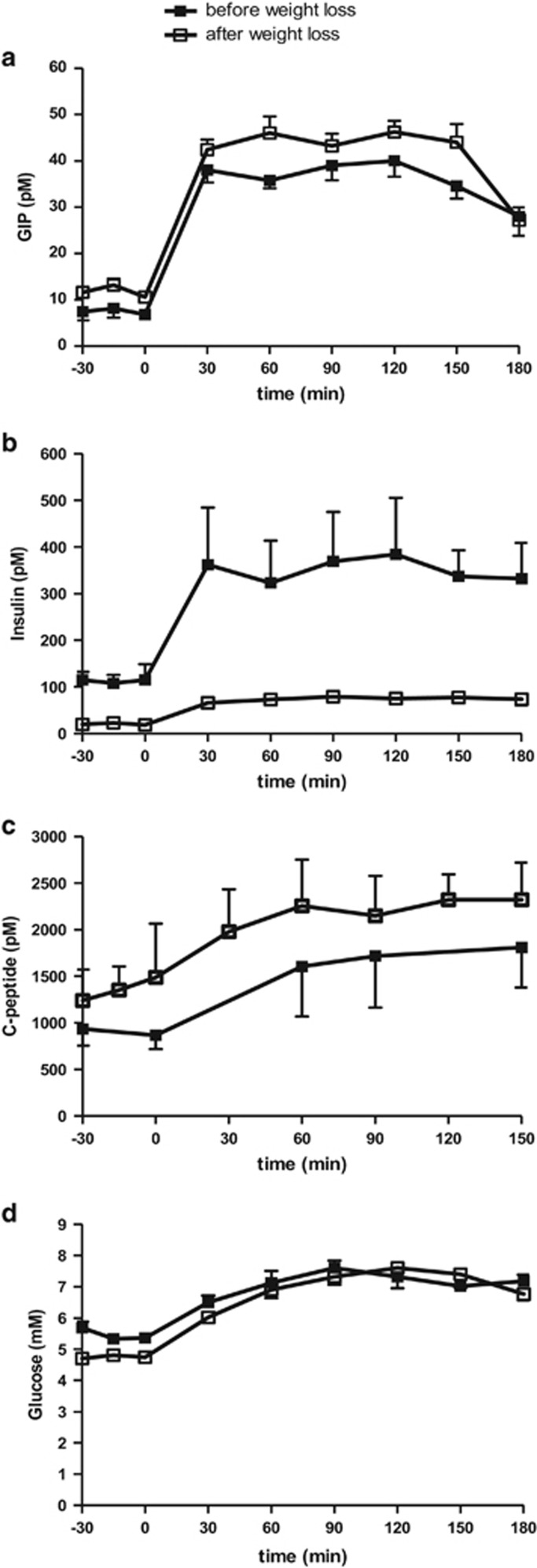
Plasma arterial GIP (**a**), insulin (**b**), C-peptide (**c**) and glucose (**d**) concentrations during GIP and HI-HG clamp before (solid squares) and after (open squares) weight loss. Data are mean±s.e.m.

Weight loss did not affect intact GIP concentrations which reached physiological postprandial plateau levels at 39.7±2.7 pm during GIP infusion and did not differ significantly from GIP plateau levels (36.9±1.8 pm) before weight loss, Figure 2a.

Insulin concentrations decreased significantly with weight loss, Figure 2b. Baseline plasma insulin concentration was significantly lower after weight loss 20.4±1.2 pm compared with insulin concentration before weight loss 112.6±2.7 pm, *P<*0.01. During the clamp period, insulin increased significantly during the first 30 min to a mean plateau level of 74.2±1.8 pm; however, the concentrations were significantly lower than before weight loss (351±9.8 pm, *P<*0.01), Figure 2b.

**Figure 3:**
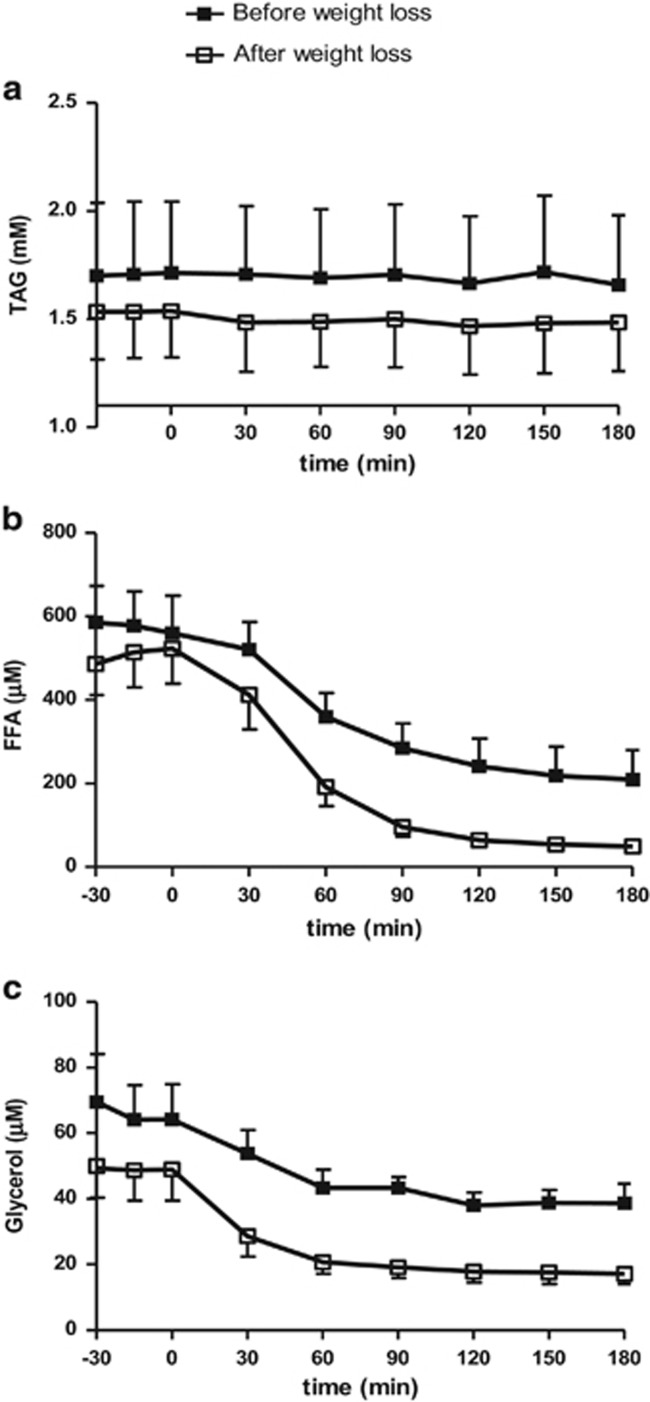
Plasma arterial TAG (**a**), FFA (**b**) and glycerol (**c**) concentrations during GIP, hyperinsulinemic and hyperglycemic clamp before (solid squares) and after (open squares) weight loss. Data are mean±s.e.m.

After weight loss, basal C-peptide concentrations were higher, however not significantly (1361±71.5 vs 901.3±34.4 pm, Figure 3c). However, during the clamp period C-peptide concentrations increased significantly during the first 30 min to a mean plateau level of 2261±31.5 after weight loss compared with before weight loss 1711±58.6 pm, *P<*0.05.

Baseline glucose levels decreased significantly with weight loss compared with before weight loss 4.7±0.02 vs 5.5±0.1 mm, *P*=0.03, Figure 3d. During GIP and the clamp period, glucose levels increased to a mean plateau level at 7±0.2 mm and were similar to glucose levels before weight loss 7.1±0.1 mm. However, a larger amount of glucose was required after weight loss (20.6±4.1 g h^−^^1^) to maintain same glucose levels as before weight loss (13.3±3.5 g h^−^^1^), *P<*0.05.

TAG concentrations were not affected by weight loss and were virtually constant throughout the study, Figure 3a. Baseline FFA concentrations decreased significantly after weight loss (507±10 vs 575±7 μm before weight loss, *P*=0.05, Figure 3b). During GIP and the clamp period, FFA concentrations declined and were significantly lower after weight loss, 144±57.8 vs 305.7±48.5 μm, *P<*0.01.

Likewise, weight loss lowered baseline glycerol concentrations (49±0.2 vs 66±1.8 μm before weight loss, *P<*0.01, Figure 3c). During GIP and the clamp period, glycerol concentrations decreased and were significantly lower after weight loss compared with glycerol concentrations before weight loss (20.1±1.8 and 42.6±2.4 μm, *P<*0.01).

### Changes in subcutaneous abdominal ATBF

**Figure 4:**
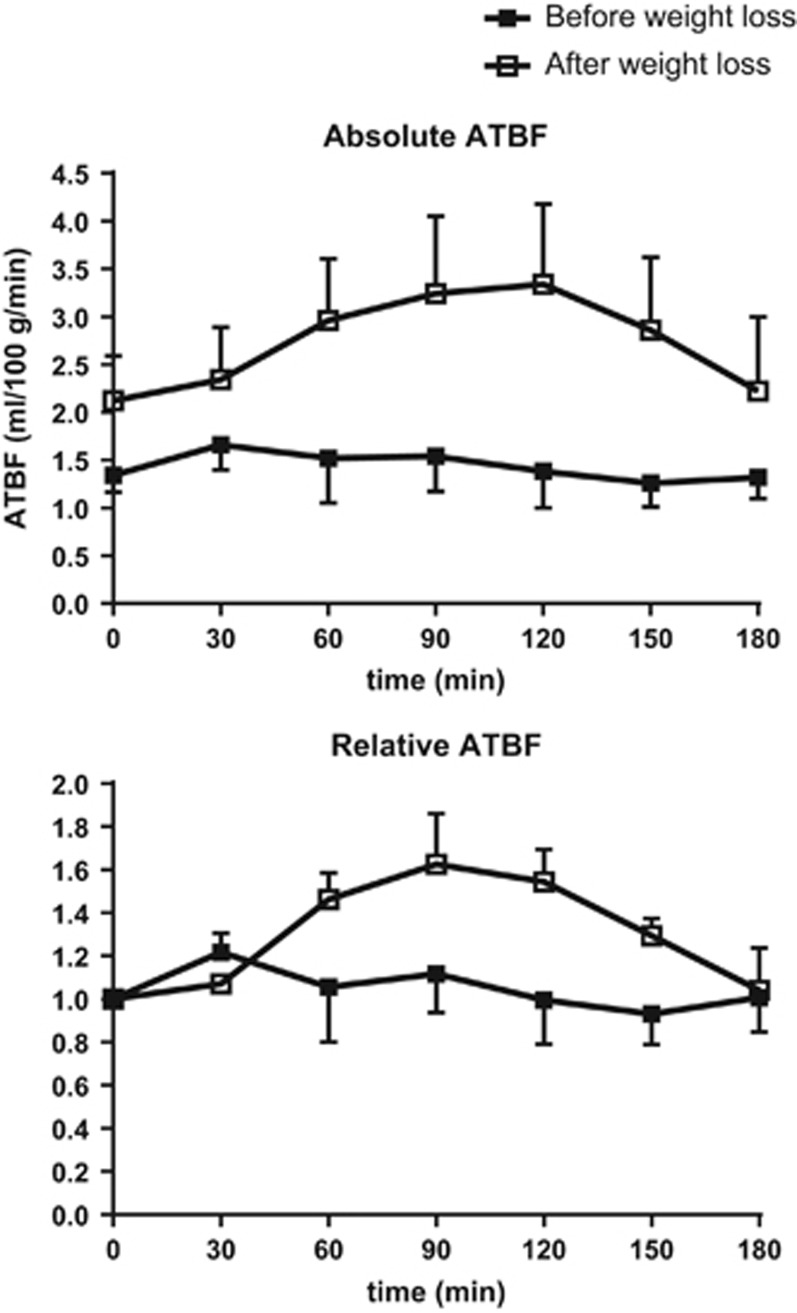
Subcutaneous abdominal ATBF during GIP, hyperinsulinemic and hyperglycemic clamp before (solid squares) and after (open squares) weight loss. Data are mean±s.e.m.

No significant differences were seen in basal ATBF before (1.3±0.4 ml min^−1^ 100 g tissue^−1^) and after weight loss (2.1±0.4 ml min^−1^ 100 g tissue)^−1^, although there was a tendency for higher values after the weight loss, *P*=0.1, Figure 4. After the weight loss, ATBF increased significantly during the clamp and GIP infusion to a plateau level at 3.2±0.1 ml^−1^ 100 g^−1^ min^−1^, *P*=0.02. After 150 min, ATBF began to reach the basal level again.

Before weight loss, ATBF remained virtually constant despite GIP infusion and clamping (1.4±0.06 ml^−1^ 100 g^−1^ min^−1^).

### Changes in net fluxes of TAG, FFA, glycerol and glucose

**Figure 5:**
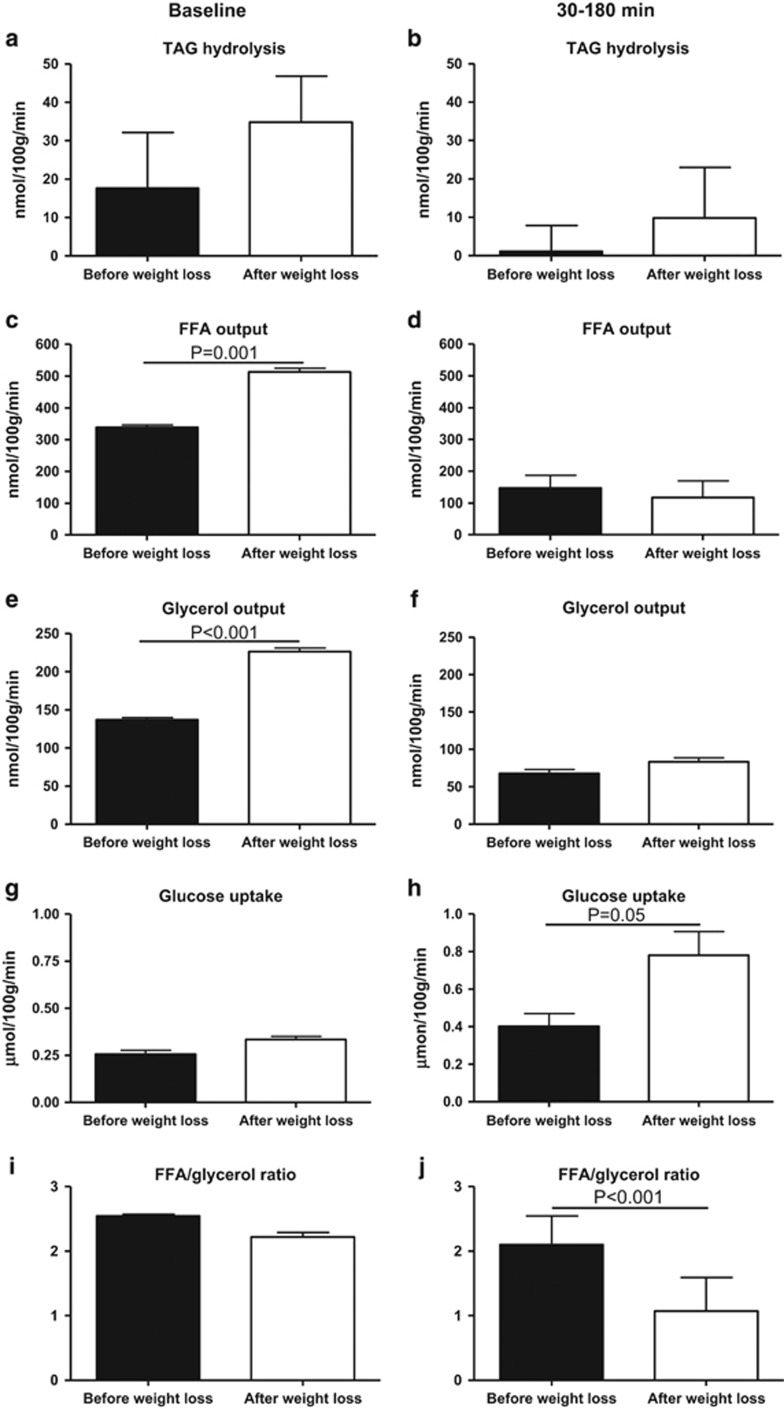
Average net fluxes of TAG, FFA, glycerol and glucose in subcutaneous abdominal adipose tissue at baseline (**a, c, e, g**) and during the steady-state period (**b, d, f, h**) from 30 to 180 min after commencement of the infusions before (black bars) and after (white bars) weight loss. Average adipose tissue FFA/glycerol output ratio at baseline (**i**) and during the steady-state period (**j**) from 30 to 180 min before (black bar) and after (white bar) weight loss. Data are mean±s.e.m.

It was not possible to demonstrate any significant TAG uptake in the adipose tissue in the baseline period or during the clamp period, Figures 5a and b.

Baseline FFA and glycerol output was significantly increased after weight loss, *P<*0.001, Figures 5c and e. During GIP and the clamp period, FFA and glycerol output declined significantly *P<*0.05, with no differences before and after weight loss, Figures 5d and f.

Weight loss did not affect baseline glucose uptake, but was associated with a significantly increased glucose uptake during the clamp period, *P*=0.05, Figures 5g and h.

Baseline FFA/glycerol ratio was not affected by weight loss, but decreased significantly during the clamp period after as opposed to before weight loss, *P<*0.001, Figure 5i and j.

## Discussion

A novel finding in the present study is that weight loss induced by caloric restriction partly restored the increase in ATBF during GIP infusion in combination with a hyperinsulinemic and hyperglycemic clamp. Simultaneously with the increase in ATBF, an increase in glucose uptake and FFA re-esterification (judging from the FFA/glycerol ratio) took place while the glycerol output was reduced to similar levels before and after weight loss, respectively. The insulin concentration after weight loss was significantly lower compared with before weight loss. This shows that weight loss rendered the subcutaneous abdominal adipose tissue more insulin sensitive with respect to both carbohydrate and lipid metabolism, which is in accord with the increase in FFA re-esterification. On the other hand, we cannot rule out that our findings could be due to the hypocaloric diet *per se*, as it has been shown in other studies that a hypocaloric diet may change glucose and insulin metabolism before a significant weight loss.^17^ However, both experiments were conducted during weight stability, that is, the energy intake was in these phases eucaloric. Although the energy intake after weight loss was significantly lower than before weight loss, we find it unlikely that this can explain the changes in ATBF in the present study. This in agreement with previous studies looking at the effect of hypocaloric diets on ATBF.^10, 18, 19, 20^

Several studies have suggested that the effect of obesity on ATBF may be a feature of insulin resistance rather than obesity *per se*.^21^ Jansson *et al.*^22^ showed a reduction in fasting ATBF and in the ATBF response to oral glucose in obese people compared with lean people with a further reduction in those with type 2 diabetes. Recently, we showed that the responsiveness of ATBF to GIP was blunted in subjects with IGT compared with subjects with NGT and lean subjects.^7^ Our present results indicate that there is a link between GIP and insulin sensitivity in subcutaneous adipose tissue in line with findings in several recent studies. A decreased expression of the GIP receptor (GIPr) in subcutaneous adipose tissue has been shown to be associated with signs of insulin resistance in non-diabetic women with central obesity.^23^ Another study indicated that GIPr expression in subcutaneous adipose tissue is inversely associated with BMI.^24^ A recent study demonstrated a defective GIP/GIPr signaling axis in adipose tissue in obese subjects.^25^ This defect was characterized by decreased GIPr expression in the subcutaneous adipose tissue, showing an inverse relationship with insulin resistance. These data suggest that the low levels of GIPr detected in obese subjects might be directly associated with the loss of insulin sensitivity associated with weight gain.

In contrast to our previous demonstration of increased TAG hydrolysis during GIP infusion, it was not possible to demonstrate any change in the TAG uptake in the present experiments. This is probably due to the high circulating TAG concentration compared with the accuracy of the applied TAG analysis rendering it difficult to detect small arterio-venous concentration differences. The ratio of glycerol to FFA release was used to estimate the extent of *in situ* FFA re-esterification. A ratio of about 3 indicates that the fatty acid derived from intracellular lipolysis is released into the circulation, whereas a ratio of ~0 indicates that the fatty acids are re-esterified and stored as TAG again.^26^ Whether the low ratio is reflecting re-esterification of FFA, derived from lipoprotein lipase-mediated hydrolysis of the circulating TAG or whether it reflects increased re-esterification of intracellular FFA cannot be determined in the present experimental setup.

The glycerol mobilization rate is usually assumed to be equal to the lipolytic rate in the tissue.^26^ The baseline glycerol output was increased after weight loss and suppressed during the clamp even in the presence of lower plasma insulin levels. This suggests improved insulin sensitivity in the adipose tissue after weight loss.

In our previous study,^7^ we demonstrated that fasting ATBF is reduced in obese subjects as shown in several previous studies.^27^ No significant difference was seen in fasting ATBF after weight loss, although there was a tendency. Thus, the magnitude of fat-mass reduction might have a role in the diet-induced effect on ATBF. No change in ATBF was found following the interventions, leading to weight loss <5% of the original body weight (11 days of very-low-calorie diet^19^ or 4 weeks of low-calorie diet^20^), whereas the improvement of ATBF was detected following 4 weeks of very-low-calorie diet, resulting in weight loss of 9% of original weight.^18^ Persistent weight loss (~40 kg) and a long period of metabolic stabilization subsequent to Roux-en Y gastric bypass in obese subjects were associated with a slight but nevertheless significant increase in ATBF, although the subjects remained overweight (BMI 28.1 kg m^−^^2^).^28^ In our study, the reduction in fat mass mainly took place in other adipose tissue compartments than the subcutaneous abdominal adipose tissue as measured on the dual-energy X-ray scans scans by counting the number of pixels from the outer body contour to the estimated outer border of the abdominal muscles. It would be interesting to investigate whether further weight loss involving also the subcutaneous abdominal adipose tissue could lead to greater ATBF improvement.

It has been shown that the initial loss of fat mass during a low-calorie diet is due to a reduction in liver fat.^29,30^ The amount of liver fat due to non-alcoholic causes increases in parallel with increasing obesity.^31^ Hyperinsulinemia appears to be the closest correlate of liver fat. Fatty liver is associated with both impaired insulin action to suppress hepatic glucose production and impaired insulin clearance.^32^ In our present study, arterial insulin concentrations increased almost fourfold during the infusion period. However, insulin concentrations were significantly lower after weight loss which is in accord with increased hepatic insulin extraction as indicated by the higher fasting C-peptide/insulin concentration ratio after weight loss.^33^

The improved insulin sensitivity after weight loss as evidenced by the lower glucose infusion rates we had to apply to induce hyperglycemia may have contributed to the improved vascular effect of GIP. Therefore, the present experiments cannot exclude that insulin or the insulin sensitivity of adipose tissue may have a permissive role in the vascular effect of GIP in adipose tissue. The higher C-peptide concentrations found during the GIP infusion may indicate an increased pancreatic sensitivity to GIP after weight loss.

In conclusion, in the weight stable phase of at least 4 weeks after weight loss induced by calorie restriction, the blunted effect of GIP on subcutaneous abdominal ATBF and metabolism is partly restored in obese subjects.

## Figures and Tables

**Table 1:** Clinical and metabolic characteristics of subjects during dietary intervention

	*Weight loss*	
	*Before*	*After*
Age (years)	42±3	43±3
Body weight (kg)	115.7±9.1	108.5±9.5*
BMI (kg m^−2^)	35±2	32±2*
Fat (%)	34.8±3.8	32.3±4*
Fat mass (kg)	38.1±6.1	34.4±6.2*
Insulin (pm)	112.6±2.4	20.4±1.7*
Glucose (mm)	5.5±0.1	4.7±0.1*
HOMA-IR	3.6±0.3	0.7±0.04*
Triacylglycerol (mm)	1.7±0.004	1.5±0.001*
FFA (μm)	574.9±7.4	507.9±10.9
Glycerol (μm)	65.9±1.8	49.1±0.2*

Abbreviations: BMI, body mass index; FFA, free fatty acid; HOMA-IR, homeostasis model assessment of insulin resistance. Values are mean±s.e.m. *P<0.01, compared with before.
